# Severe thiamine deficiency in eastern Baltic cod (*Gadus morhua*)

**DOI:** 10.1371/journal.pone.0227201

**Published:** 2020-01-02

**Authors:** Josefin Engelhardt, Oscar Frisell, Hanna Gustavsson, Tomas Hansson, Rajlie Sjöberg, Tracy K. Collier, Lennart Balk

**Affiliations:** 1 Department of Environmental Science and Analytical Chemistry, Stockholm University, Stockholm, Sweden; 2 Institute of Marine Research, Swedish University of Agricultural Sciences, Lysekil, Sweden; 3 Huxley College of the Environment, Western Washington University, Bellingham, Washington, United States of America; University of Siena, ITALY

## Abstract

The eastern Baltic cod (*Gadus morhua*) population has been decreasing in the Baltic Sea for at least 30 years. Condition indices of the Baltic cod have decreased, and previous studies have suggested that this might be due to overfishing, predation, lower dissolved oxygen or changes in salinity. However, numerous studies from the Baltic Sea have demonstrated an ongoing thiamine deficiency in several animal classes, both invertebrates and vertebrates. The thiamine status of the eastern Baltic cod was investigated to determine if thiamine deficiency might be a factor in ongoing population declines. Thiamine concentrations were determined by chemical analyses of thiamine, thiamine monophosphate and thiamine diphosphate (combined SumT) in the liver using high performance liquid chromatography. Biochemical analyses measured the activity of the thiamine diphosphate-dependent enzyme transketolase to determine the proportion of apoenzymes in both liver and brain tissue. These biochemical analyses showed that 77% of the cod were thiamine deficient in the liver, of which 13% had a severe thiamine deficiency (*i*.*e*. 25% transketolase enzymes lacked thiamine diphosphate). The brain tissue of 77% of the cod showed thiamine deficiency, of which 64% showed severe thiamine deficiency. The thiamine deficiency biomarkers were investigated to find correlations to different biological parameters, such as length, weight, otolith weight, age (annuli counting) and different organ weights. The results suggested that thiamine deficiency increased with age. The SumT concentration ranged between 2.4–24 nmol/g in the liver, where the specimens with heavier otoliths had lower values of SumT (*P* = 0.0031). Of the cod sampled, only 2% of the specimens had a Fulton’s condition factor indicating a healthy specimen, and 49% had a condition factor below 0.8, indicating poor health status. These results, showing a severe thiamine deficiency in eastern Baltic cod from the only known area where spawning presently occurs for this species, are of grave concern.

## Introduction

Thiamine, vitamin B_1_, is a water-soluble molecule consisting of a thiazole ring linked by a methylene bridge to a pyrimidine ring. Thiamine is primarily produced by plants, but also by some fungi and bacteria [1]. When an animal cell receives thiamine (T), it will be converted to thiamine diphosphate (TDP) by the enzyme thiamine pyrophosphokinase [2]. Thiamine monophosphate (TMP) is mainly regarded as a degradation product which will be recycled or excreted. TDP functions as a cofactor to at least five different vital enzymes in cell metabolism. One of them is branched-chain α-keto acid dehydrogenase, active in the metabolism of branched amino acids [3, 4]. Another TDP-dependent enzyme is 2-hydroxyphytanoyl-CoA lyase, involved in the α-oxidation of certain fatty acids [5, 6]. These fatty acids are degradation products of chlorophyll and are degraded to formyl-CoA [6]. However, if the enzyme is lacking TDP, in case of thiamine deficiency, phytanic acid is formed instead [6]. Transketolase (TK), in the hexose monophosphate shunt [7, 8] is also a TDP-dependent enzyme, and is a provider of nucleic acid bases, used as the backbone for DNA and RNA, and NADPH used as a protection against oxidation and in the synthesis of fatty acids [9]. Another TDP-dependent enzyme, pyruvate dehydrogenase, acts as the bridge between glycolysis and the TCA cycle [9–11]. The enzyme converts pyruvate to acetyl-CoA, thus in the case of thiamine deficiency, the concentration of pyruvate is likely to increase. The accumulated pyruvate will then be converted to lactate instead, which in high concentration is toxic to the organism [12]. Lastly, α-ketoglutarate dehydrogenase, active in the TCA cycle, is also TDP dependent [13, 14]. During the reaction of the enzyme, NADH is formed, used in the electron transport chain to produce ATP [9]. One common denominator of some of these enzymes is the production of acetyl-CoA. During thiamine deficiency, a way for the cell to compensate for the decrease in concentration of acetyl-CoA is oxidation of fatty acids, and this could be one reason for why a thiamine deficient animal becomes emaciated. Thiamine is an essential vitamin for animals acquired from food and a deficiency of thiamine is often lethal [15, 16]. Previous studies have concluded that thiamine homeostasis in the brain is prioritized over homeostasis in other organs [17]. In the absence of thiamine, toxic substances such as glyoxals [18], lactate [19] and phytanic acid [20–22] can form and accumulate. Overall, thiamine deficiency leads to disruption of the metabolism of carbohydrates, lipids and proteins. Because thiamine acts as a cofactor to enzymes in several different pathways, and a deficiency causes an imbalance of metabolites, the sub-lethal symptoms of thiamine deficiency are diverse and, depending on animal class, can include emaciation, memory loss, neurological disorders, immunosuppression, reduced vision and sense of smell, hypothermia, degradation of the blood-brain barrier, anorexia, labored breathing as well as memory-, learning-, orientation-, behaviour- and reproductive disorders [12, 23–28].

Thiamine deficiency was first detected in wild species of salmon by Fitzsimons in 1995 [29], since then the deficiency has been observed among numerous different wild animal species. The reproductive disorder observed in salmon, where eggs and larvae die, was proven to be specifically caused by a deficiency of thiamine [15, 29].

Birds suffering from thiamine deficiency are common black-headed gull (*Chroicocephalus ridibundus*), common eider (*Somateria mollissima*), common starling (*Sturnus vulgaris*), great black-backed gull (*Larus marinus*), herring gull (*Larus argentatus*), hooded crow (*Corvus cornix*) [16] and red wattlebird (*Anthochaera carunculata*) [30]. Fish observed with thiamine deficiency are American eel (*Anguilla rostrata*) [31, 32], Atlantic salmon (*Salmo salar*) [32, 33], Chinook salmon (*Oncorhynchus tshawytscha*) [34], Coho salmon (*Oncorhynchus kisutch*) [35], European eel (*Anguilla anguilla*) [32], lake trout (*Salvelinus namaycush*) [35–37], sea trout (*Salmo trutta*) [33] and steelhead trout (*Oncorhynchus mykiss*) [35]. Thiamine deficiency has also been observed in blue mussels (*Mytilus sp*.) [32] and in American alligators (*Alligator mississippiensis*) [38]. Due to the diversity of affected species, thiamine deficiency has recently been hypothesized to be a possible driver of wildlife population declines [39].

The Baltic Sea cod (*Gadus morhua*) have been divided into three different populations defined by spawning areas, where the eastern Baltic (EB) cod has its spawning grounds east of Hanöbukten Bay, the western Baltic cod spawn in Öresund and another population belonging to the North Sea spawns outside the Swedish west coast. Historically, the EB cod population spawned in three locations, the Bornholm Basin, the Gdansk Deep and the Gotland Basin [40]. The Bornholm Basin, in the Hanöbukten Bay, is the only important spawning area left for the EB cod today. Some suggested reasons for the loss of spawning locations are changes in dissolved oxygen and salinity in these areas, which are dependent on the low inflow of marine water from the Atlantic ocean through the narrow inlet into the Baltic Sea [41]. The EB cod population has decreased drastically over the past three decades. The total catch reported to the International Council for the Exploration of the Sea (ICES) in 2017 was the lowest observed value since the record started in 1965 [42]. ICES has recently advised a zero catch in 2020 [43]. The total commercial harvest of cod in Sweden has declined from 20,000 tonnes in 2001 to 2,500 tonnes in 2018 [44]. The condition indices of the EB cod have decreased as well and the size range of the population is truncated [43, 45–47]. The size at first maturation has decreased by almost 40% within 20 years, from a length of around 40 cm in the 1990s to around 25 cm in the 2010s [41]. The spawning period has changed but the reasons why are not known [48, 49]. Furthermore, the survival rate of the egg and larval stages of the EB cod population seems to be highly variable [41]. Suggested explanations for the EB cod’s decline are many, but among others, overfishing [33], low availability of prey [50], altered metabolism due to the spread of low oxygen areas [51], reduced food uptake [52–54], increased parasitic loads [55], size-selective commercial fishing [56] leading to fisheries-induced evolution [57], lower salinity and oxygen levels [58–60], predation of cod eggs [61] and lowered reproductive success in the Baltic Sea [62]. Nematode infestations in the EB cod, mainly by *Contracaecum osculatum*, are assumed to be associated with the rising occurrence of gray seal (*Halichoerus grypus*), the final host, in the spawning area [63, 64]. These nematodes are suggested to affect the survival of the EB cod [65].

The thiamine concentration in EB cod from the Baltic Proper has, to our knowledge, only been analyzed once [33]. In that study, the thiamine concentration in liver and gonad tissues were determined, but no biochemical analysis was done. The study concluded that further analysis was needed to determine the thiamine status of the EB cod [33]. The thiamine status of a specimen is best investigated through a combination of biochemical and analytical chemistry analysis. The biochemical measurements show the specific activity and the proportion of apoenzymes (enzymes without TDP), yielding the latency of the enzyme [66]. In this study, latency specifically refers to the proportion of the enzyme transketolase not bound to TDP (apoenzymes). When determining the latency of a sample, the endogenous activity is first determined, by measuring the enzyme activity of the sample. The endogenous activity is the activity corresponding to the proportion of enzymes with TDP (holoenzymes). Thereafter, the cofactor TDP is added in excess and can bind to the enzyme, and the maximum activity is determined. When both the endogenous and the maximum activity has been determined, the latency can be calculated according to Eq 1. The latency should theoretically be zero in a healthy individual, and consequently the endogenous and maximum activity should be the same.

$$Latency\;[\%]=(\frac{maximum\;activity-endogeneous\;activity}{maximum\;activity})\times 100$$ (1)

Analytical chemical measurements with high performance liquid chromatography (HPLC) can measure the concentrations of T, TMP and TDP (combined SumT). Every time a new tissue is chemically analyzed, the concentration needs to be evaluated in relation to the specific activity and latency to understand whether or not the tissue is thiamine deficient. The aim of this current study is to determine the thiamine status of the EB cod. The relation between the biochemical and chemical analysis will be established in this study, allowing for comparison with the thiamine status from twenty years ago [33].

## Materials and methods

### Sampling

**Fig 1:**
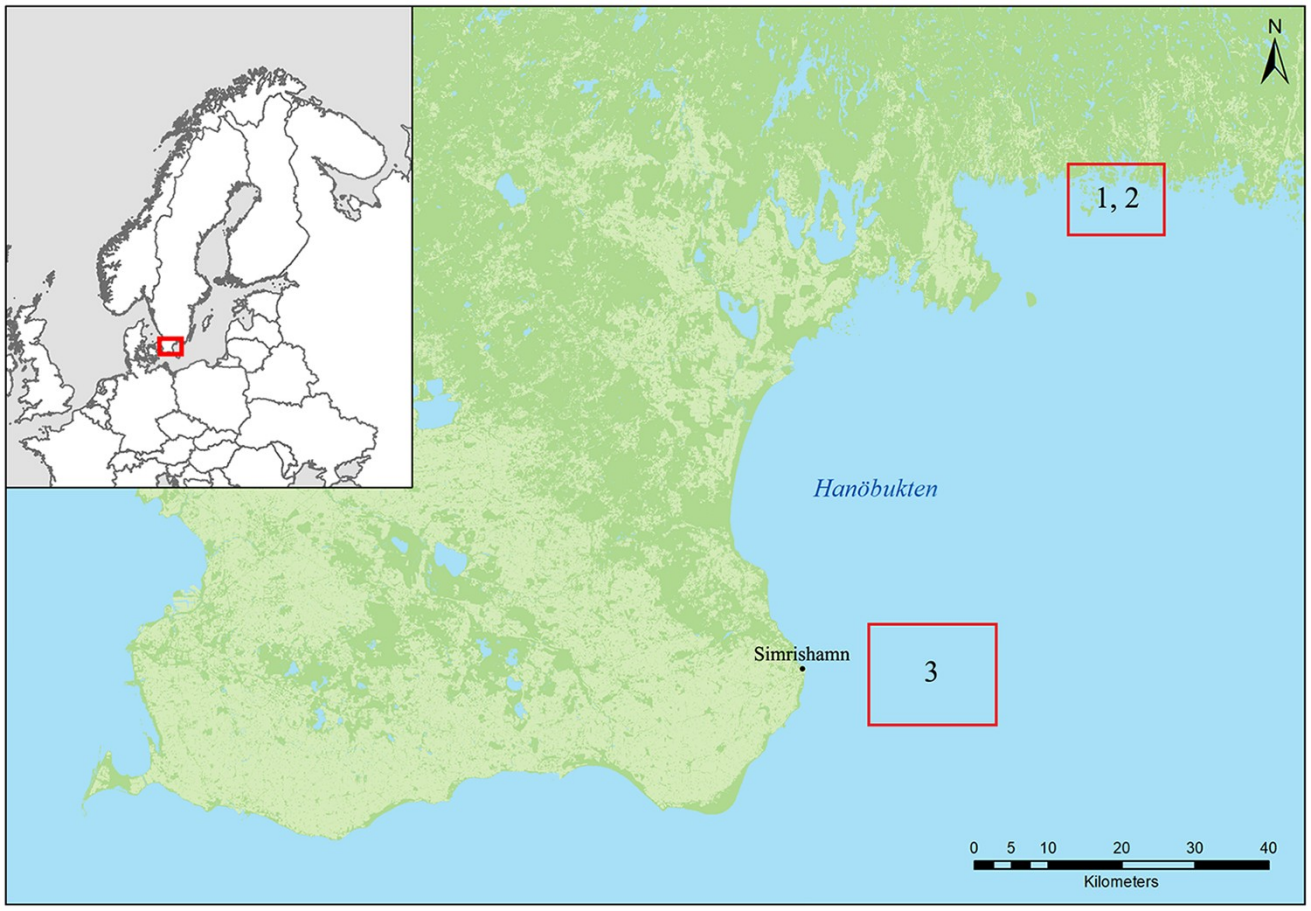
Sampling area in Hanöbukten Bay in the Bornholm Basin. Batch 1 and 2 were caught in the upper square, and batch 3 at Långagrund outside Simrishamn, presented in the lower square.

The EB cod used in this study originate from the Baltic Sea, in ICES subdivision 25 [67]. The EB cod were caught using a fish trap or with a spinning rod with a jig in Hanöbukten Bay between the 23rd of October and 4th of November 2017. A total of 51 EB cod were caught and divided into three batches. Batch 1 and 2 were caught using a push-up trap while batch 3 was caught using a spinning rod with a jig. Most of batch 1 and 2 were caught at the coordinates 56°8.34’N15°3.14’E, and some at the coordinates 56°8.30’N15°1.80’E (Fig 1). Batch 3 was caught at 30 m depth at Långagrund outside Simrishamn (55°33'N14°36'E) (Fig 1). After catch, the specimens were put in a fish tank with aerated water from the collection site, and transported to the laboratory for sampling. The tank water was replaced every 24 hours. Each specimen was stunned by a blow on the head and killed by cutting the spine at the neck. The specimen was weighed to the nearest 10 g and total length was measured to the nearest 1 cm. Any exterior injuries or other abnormalities were recorded. Each specimen was photographed with high resolution in left and right profile. Liver, gonad, heart and brain were dissected and weighed to the nearest 0.01 g. The sex was determined by the morphology of the gonad. Interior injuries and other abnormalities were recorded. The otoliths were dissected and later cleaned and dried at room temperature in a fume hood. The otoliths were thereafter weighed separately to the nearest 0.001 g, and the average weights of the two otholiths were calculated.

For analysis of TDP-dependent enzymes, a central piece, approximately 3 g, of the liver and the entire brain, was cut into smaller pieces with a pair of scissors and homogenized in an equal volume ice-cold 0.25 M sucrose in a 10 mL Potter-Elvehjem homogenizer (size 21) with five up and down strokes at 400 rpm and under continuous cooling with ice-water. The homogenate was diluted to 20% with ice-cold 0.25 M sucrose, transferred to 2 mL Eppendorf tubes, and centrifuged at 10,000 gav at 4°C for 10 min in an Eppendorf 5415R centrifuge (Eppendorf, Hamburg, Germany). The supernatant was carefully collected with a pipette, without unsettling the pellet, and aliquots of the supernatant were put in cryotubes, snap-frozen in liquid nitrogen, and later stored at -140°C until analysis of the TK activity. The methods used for fish sampling were approved by the Stockholm Northern Research Ethics Committee (Dnr. N209/14).

### Age determination

Recently, it has been difficult to determine the age by annuli counting of the otoliths in the EB cod [46]. The otoliths were divided through the *Sulsus acusticus*, by hand. The divided otolith parts were placed in clay with the line of cleavage facing upwards. The parts were dabbed with water and examined using a stereo microscope. The annuli were then counted to determine the age [68]. The quality of the annuli counting was scaled from 1–4 by the same person, where 1 was good, 2 was moderate (age +/- 1 year), 3 was poor (age +/- 1 year or more) and 4 was unreadable.

### Chemicals

Biochemical analysis: Bovine serum albumin (A4378), α-glycerophosphate dehydrogenase and triosephosphate isomerase (G1881), MgCl_2_ (Ultra M2670), NADH (N8129), D-ribose 5-phosphate (R7750), sucrose (Ultra S7903), thiamine diphosphate (C8754), Tris-HCl (T3253), D-xylulose 5-phosphate (15807) were purchased from Sigma Aldrich Sweden AB. Copper sulphate *p*.*a*. (1.02791.0250), disodium carbonate *p*.*a*. (1.06392.1000), Folin-Ciocalteu’s phenol reagent (1.09001.0500), hydrochloric acid 30% Suprapur (1.00318.1000), potassium sodium tartrate *p*.*a*. (1.08087.500) were purchased from Merck (Darmstadt, Germany). Sodium hydroxide, Baker-analyzed (0402) was purchased from J. T. Baker (Deventer, the Netherlands). Chemical analysis: Thiamine (47858), thiamine monophosphate (T8637) and thiamine pyrophosphate (PHR1369) were purchased from Sigma Aldrich Sweden AB. Acetonitrile LiChrosolv (1.14291.2500), dipotassium hydrogen phosphate EMSURE (1.05099.1000), potassium dihydrogen phosphate EMSURE (1.04873.1000), potassium hexacyanoferrate (III) *p*.*a*. (1.04973.0250), n-Hexan LiChrosolv (1.04391.2500), hydrochloric acid 30% Suprapur (1.00318.1000) and trichloroacetic acid *p*.*a*. (1.00807.1000) were purchased from Merck (Darmstadt, Germany). Ethyl acetate, Baker analyzed for analysis (9282–03) and sodium hydroxide, Baker-analyzed (0402) were purchased from J. T. Baker (Deventer, the Netherlands). The water used both for biochemical and chemical analysis was purified using a Milli-Q Integral 3 system (Thermo Fisher Scientific Inc., Waltham, MA, USA).

### Transketolase activity assay

The activity of transketolase was measured according to Tate and Nixon (1987) [69]. The biochemical measurements were either done on a brain or liver sample. The supernatant, stored at -140°C was thawed quickly and kept on ice. A solution with the final concentrations of 100 mM Tris-HCl, 1.20 mM MgCl_2_, 0.200 mM NADH, 7.92 U/mL α-glycerophosphate dehydrogenase and 79.2 U/mL triosephosphate isomerase, were added to the cuvette together with pure water, 0.100 M sucrose and 50 μL sample. When measuring the maximum activity, 0.100 mM TDP was added as well. The cuvette was incubated for 3 minutes, in a 30°C water bath and then run for 3 minutes, measuring at 340 nm in the spectrophotometer, as a background. For analysis, a Shimadzu 2600 UV-Vis spectrophotometer (Shimadzu Corporation, Kyoto, Japan) was used together with the software UV-Probe from the same manufacturer. Thereafter, 0.800 mM D-xylulose 5-phosphate and 10.0 mM D-ribose 5-phosphate was added to start the reaction and run for 3 additional minutes. Duplicates were made for both the endogenous and maximum activities. The activity was checked to be linear with both protein used and time of incubation.

A standard was used to ensure repetitive analytical results, between the days of enzyme activity measurements. The standard was a cod liver supernatant prepared, mixed and collected into several frozen aliquotes. The standard was treated the same way as the other samples but analyzed three times during the laboratory period, before, in the middle and after all the samples. The standard showed that the results were not affected by the time or the order of the measurements.

Protein concentrations in the samples were measured according to the method by Lowry *et al*. [70] using bovine serum albumin as the standard protein. Each liver and brain sample was diluted to be within the linear interval of the standard curve.

### Chemical analysis of thiamine

T, TMP and TDP were quantified using HPLC with fluorescence detection. The preparation and analysis of the liver samples were performed according to Brown *et al*. [71], with adjustments suggested by Kankaanpää *et al*. [72], Mörner *et al*. [73] and with the optimization that the derivatization reagent, potassium hexacyanoferrate, was prepared to a concentration of 0.2% with NaOH. Because cod livers have a high concentration of fat, the liver samples were centrifuged for up to 75 min instead of 15 min to improve the analysis. Since fat precipitated after two washing steps, five extractions were used instead of the usual three to protect the column against trichloroacetic acid and fat. A few samples were only extracted three times, but no significant difference could be seen between washing three or five times. Liver samples weighed approximately 200 mg.

### Data analysis

The statistical analysis included the Pearson correlation and t-test (two samples, assuming equal variances). An ordinary least square regression line was used in the graphs. When the correlation was not linear, the Spearman rank correlation was used instead. Only 2-tailed tests were used. *P*-values below 0.05 were considered significant and *P*-values below 0.1 were considered to have a tendency of significance. The 95% confidence intervals for the arithmetic mean are based on the t-distribution. The Shapiro-Wilk normality test and histograms were used to ensure that the assumptions of normality were met. Only biological (not technical) replicates were used, *i*.*e*. the number of observations corresponds to the number of specimens analyzed. No statistical calculations were used to determine suitable sample sizes. Instead these were based on general experience of the investigated species. Partial blinding was applied, because complete blinding was not possible.

Latency is a term with a theoretical value between 0 and 100%. However, there is always a risk of a small imprecisions in laboratory practices. Even though latency should not occur normally in tissues from healthy wild animals, values up to 6% latency can be due to imprecisions in laboratory practices, according to previous studies [32]. With the same reasoning, there can be a negative latency. In those cases, the assumed correct value should lie around 0% latency. Thus in statistical analyses of the data, all the latency values were included, even those below 0, but in the graphs the negative values were truncated. A specimen with a TK latency above 6% is considered to be thiamine deficient [32]. In this study, severe thiamine deficiency was defined in a tissue where at least one out of four enzymes are apoenzymes (≥25% latency). The cod were caught during late October, early November, not close to the spawning, which for the EB cod occurs during the summer [49]. Therefore, the total weight and the somatic weight were almost the same. The total weight was used in this study, as well as the Fulton’s condition factor (CF, Eq 2) including the total weight instead of somatic condition index (SCI, Eq 3) [74].

$$CF=\frac{W}{L^{3}}\times f$$ (2)

Where CF is the Fulton’s condition factor, W is the total weight in g, L is the total length in cm and *f* is a scaling factor, in this case 100 [74].

$$SCI=\frac{W_{s}}{L^{3}}\times f$$ (3)

Where SCI is the somatic condition factor, W_s_ is the somatic weight in g, the gonad weight subtracted from the total weight, L is the total length in cm and *f* is a scaling factor, in this case 100 [74]. However, if the cod were to be caught during spawning, somatic weight and SCI should be used. The use of the total weight could in those cases lead to assumptions of a better condition than what is true.

The female and male cod were statistically analyzed separately and together for each variable. In the cases of no difference between the sexes, female and male data were pooled in the graphic and statistical analysis. During the statistical analysis, the three batches were analyzed separately and together. The batches did not differ significantly in the different variables, such as liver proportion T, TMP, TDP, liver and brain TK latency and TK activity. The batches were therefore pooled in both the calculations and the graphs presented here.

## Results & discussion

### Characterization of the sampled material

**Fig 2:**
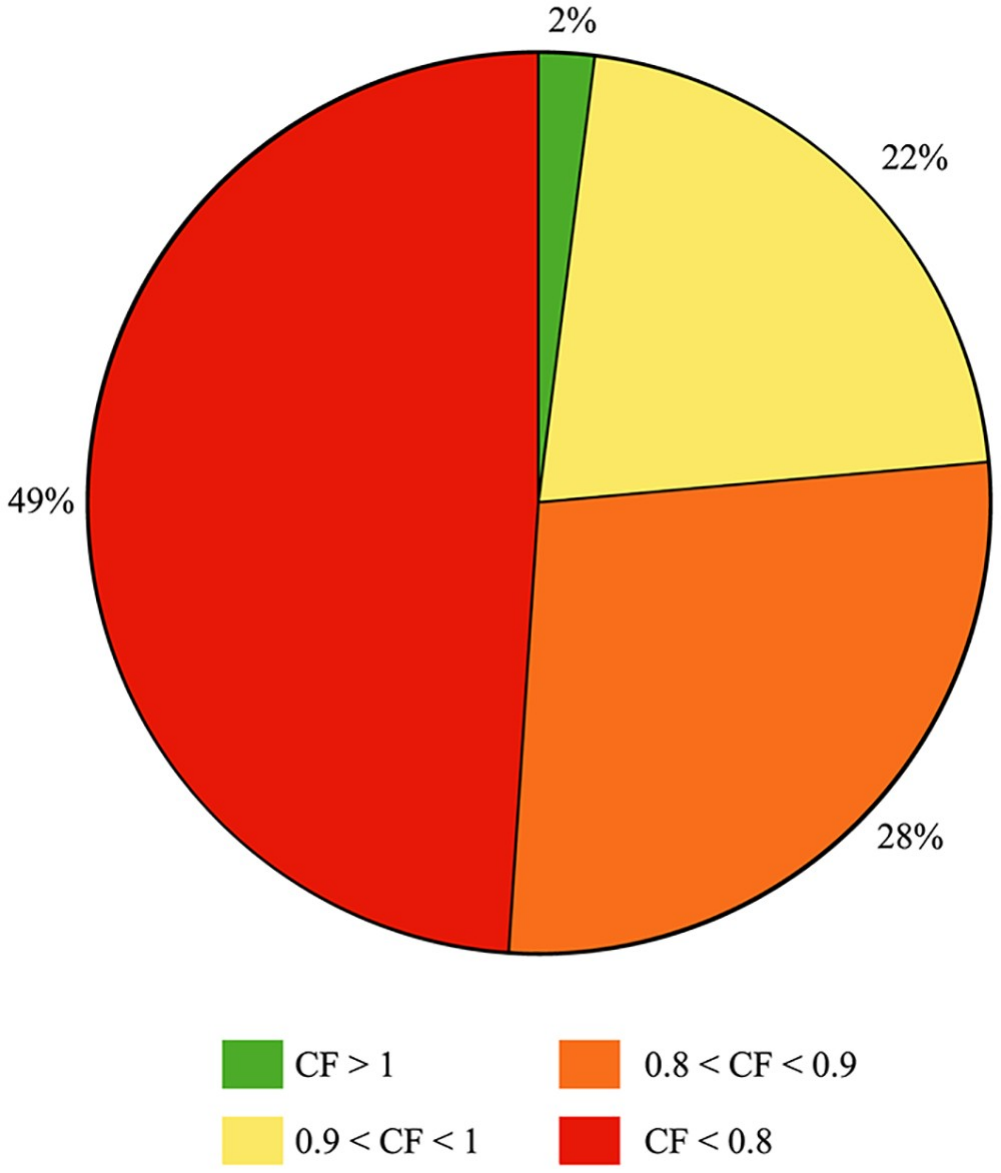
The proportion of specimens with different Fulton’s condition factor (CF). There are 98% of the specimens that have a CF value below 1 and 49% of the specimens have a CF value below 0.8.

**Fig 3:**
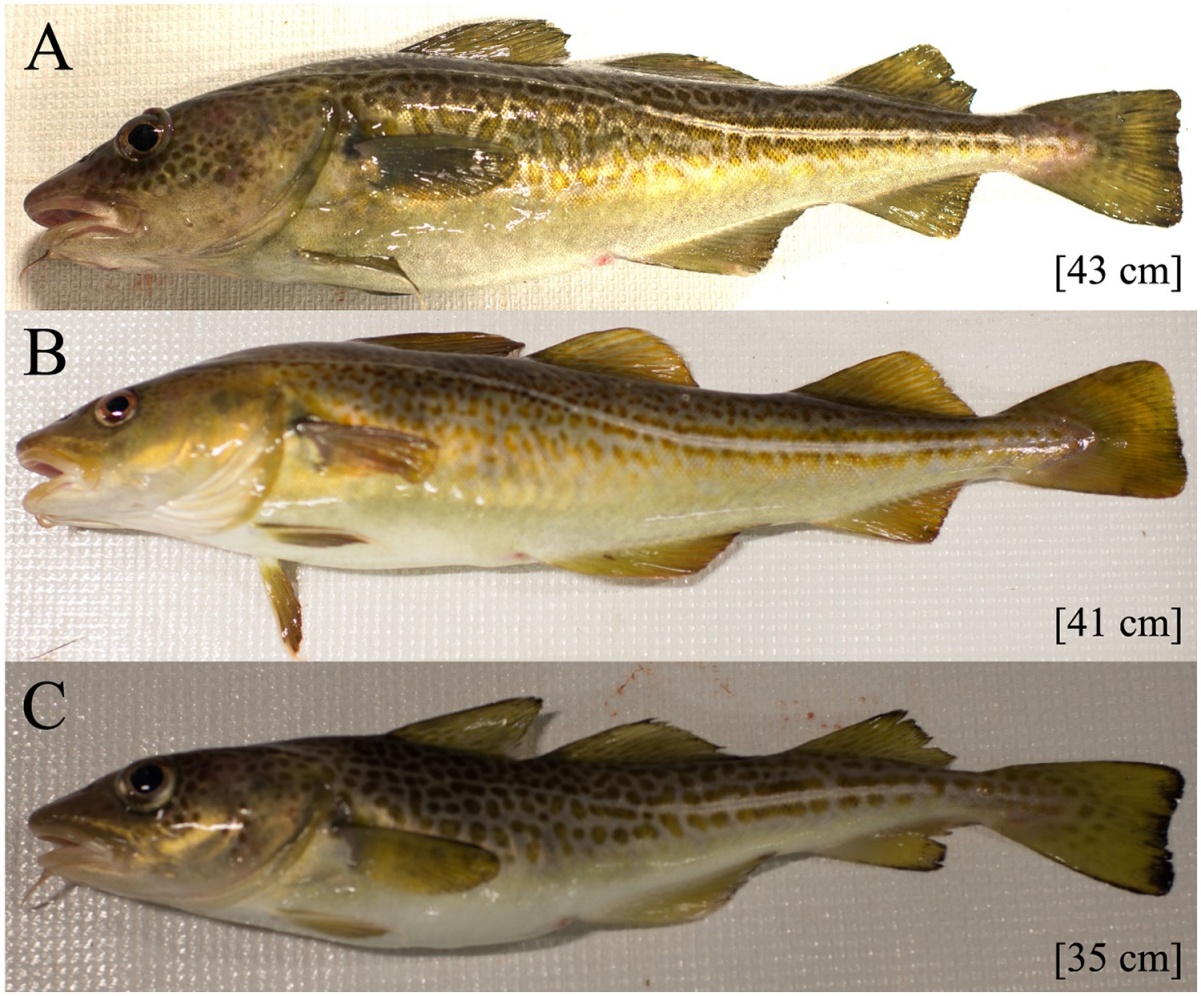
Cod with different Fulton’s condition factors. Age was determined with annuli counting of the otolith. The total length of the specimen is presented in the brackets (A) A cod with CF = 0.95, age 6 years (B) A cod with CF = 0.84, age 2 years (C) The cod with the lowest CF = 0.61, age 3 years.

The studied EB cod group, consisted of 51 specimens caught at different sites in Hanöbukten Bay during late October and early November 2017. In the studied EB cod group, 57% were female, 35% were male and the sex of 8% of the specimens were visually unidentifiable. The mean length of the specimens was 39.3 cm (range 23.1–56.5 cm). The mean total weight was 529 g (range 110–1530 g). The mean somatic weight was 522 g (range 110–1510 g). The mean Fulton’s condition factor was 0.803 (range 0.605–1.05). The mean somatic condition factor was 0.793 (range 0.589–1.04). The mean liver somatic index (LSI) was 3.71% (range 1.15–6.89%). The mean liver weight was 22.1 g (range 1.80–104 g). The mean brain weight was 0.720 g (range 0.310–1.20 g). The average weight of the two otoliths was 232 mg (range 65.0–399 mg). The average otholith weight was used for the data analysis. The mean age by annuli counting was 3.8 years (range 1–6 years). The mean quality of the annuli counting was 1.9 (range 1–3). Condition indices were consistently low in this study and were comparable to previously observed values in the Baltic Sea. Between 1987 and 1996, the EB cod CF ranged from 1.1–1.2 in the same area [75], values that may be considered as normal healthy control values. In our study, only 2% of the EB cod sampled had a CF above 1 (Fig 2). A previous study has shown that CF values below 0.9 from EB cod from the Bornholm Basin were outside the standard deviation in 2000 [46]. The current study found that 76.5% of the cod had a CF value below 0.9. Furthermore, 49% of the studied group had a CF value below 0.8. This can be compared to less than 30% of cod with a CF value below 0.8 in 2014 [46]. The specimens in this study are small and ill-proportioned (Fig 3), in which specimens with different CF values are shown. Most of the biological data were acquired for all specimens (51), except for otoliths where one was missing (50), and thiamine data, where biochemical measurements were done on 30 liver samples and 22 brain samples, and chemical measurements were done on 38 liver samples.

**Fig 4:**
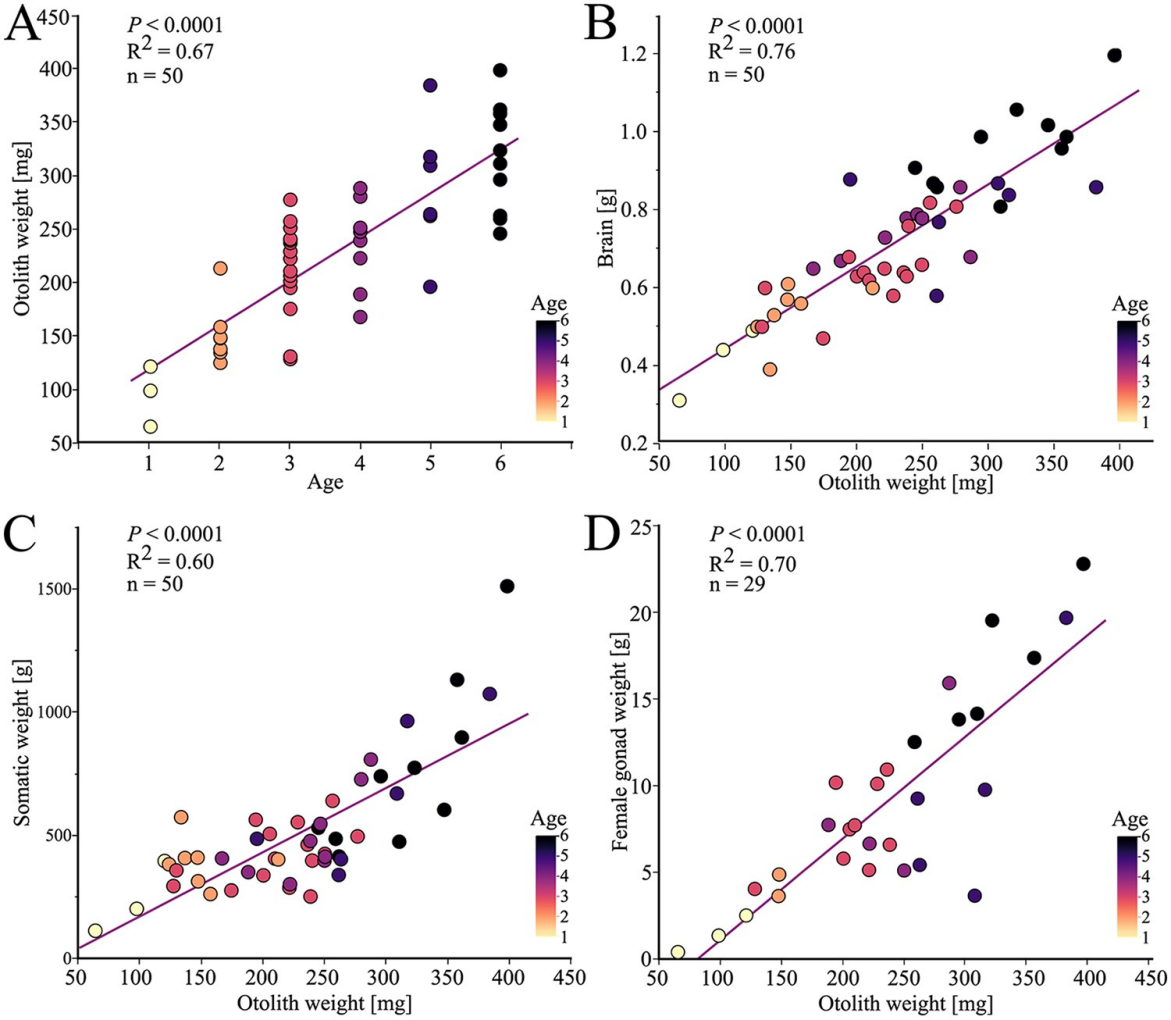
Variables describing age and growth in the investigated EB cod from the Hanöbukten Bay. Female and male EB cod were pooled (A-C). The colors correspond to the age of the specimen, determined by annuli counting of the otoliths. (A) The otolith weight increased with increasing age determined by annuli counting of the otoliths. (B) The brain weight increased with increasing otolith weight. (C) The somatic weight increased with increasing otolith weight. (D) The female gonad weight increased with increasing otolith weight.

The age by annuli counting of the EB cod is difficult to determine [76]. Different variables were plotted against each other to find the best variable corresponding to the age by annuli counting. The otolith weight correlated strongly with the age by annuli counting (*P*<0.0001, n = 50, Fig 4A), a phenomenon observed among many teleost fish species [77–82]. The growth of the brain is one of the most stable variables for an animal suffering from thiamine deficiency [83]. The growth of the brain can be considered relatively constant as an allometric standard representative for the age of the cod (Fig 4B). The brain weight increased with increasing age (*P*<0.0001, n = 50, represented by colors in Fig 4B) and with increasing otolith weight (*P*<0.0001, n = 50, Fig 4B). The somatic weight increased with increasing age (*P*<0.0001, n = 50, represented by colors in Fig 4C) and with the otolith weight (*P*<0.0001, n = 50, Fig 4C). It can be assumed that the best way to estimate the age of the cod in this study was through the brain weight or the otolith weight. According to previous studies regarding otolith weight from cod, the cod in this study were assumed to range in age from 1–6 years old [82]. The age by annuli counting of the cod corresponded to exactly the same range, *i*.*e*. 1–6 years old (Fig 4A). The female gonad weight increased with increasing age (*P*<0.0001, n = 29, represented by colors in Fig 4D) and with the otolith weight (*P*<0.0001, n = 29, Fig 4D). There were a few female specimens that did not follow the trend of increasing the gonad weight as the otolith weight increases. It could be that these specimens are not able to mature properly, however further investigations more closely to the spawning time period should be made before conclusions can be drawn. There was no correlation between the LSI and gonad somatic index in females (*P* = 0.88, n = 29, not shown), indicating that the gonad has not started to mature.

### Evidence of severe thiamine deficiency in liver and brain

**Fig 5:**
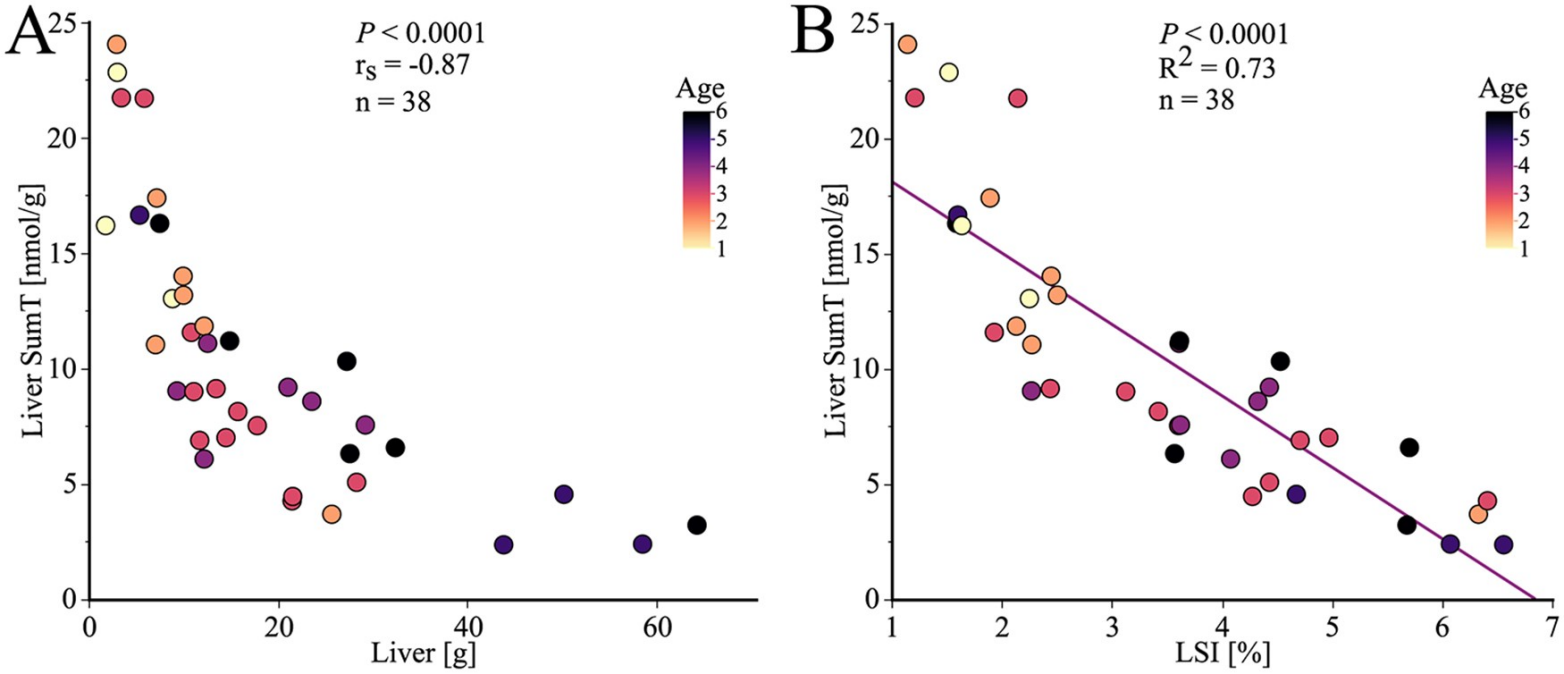
Relationship between liver SumT concentration and liver weight. The colors correspond to the age of each specimen, determined by annuli counting of the otoliths. (A) The concentration of T, TMP and TDP (combined SumT) decreased with increasing liver weight (Spearman correlation). (B) The SumT concentration decreased with increasing relative liver somatic weight (LSI).

The concentration of liver SumT decreased as liver size increased (*P*<0.0001, n = 38, Fig 5A). The SumT decreased with increasing age (*P* = 0.017, n = 38, as shown in Fig 5A). The liver weight increased with increasing age (*P*<0.0001, n = 50, represented by colors in Fig 5A). The concentration of liver SumT decreased with increasing relative size of the liver, LSI (*P*<0.0001, n = 38, Fig 5B). The LSI increased with increasing age (*P* = 0.0041, n = 50, represented by colors in Fig 5B).

**Fig 6:**
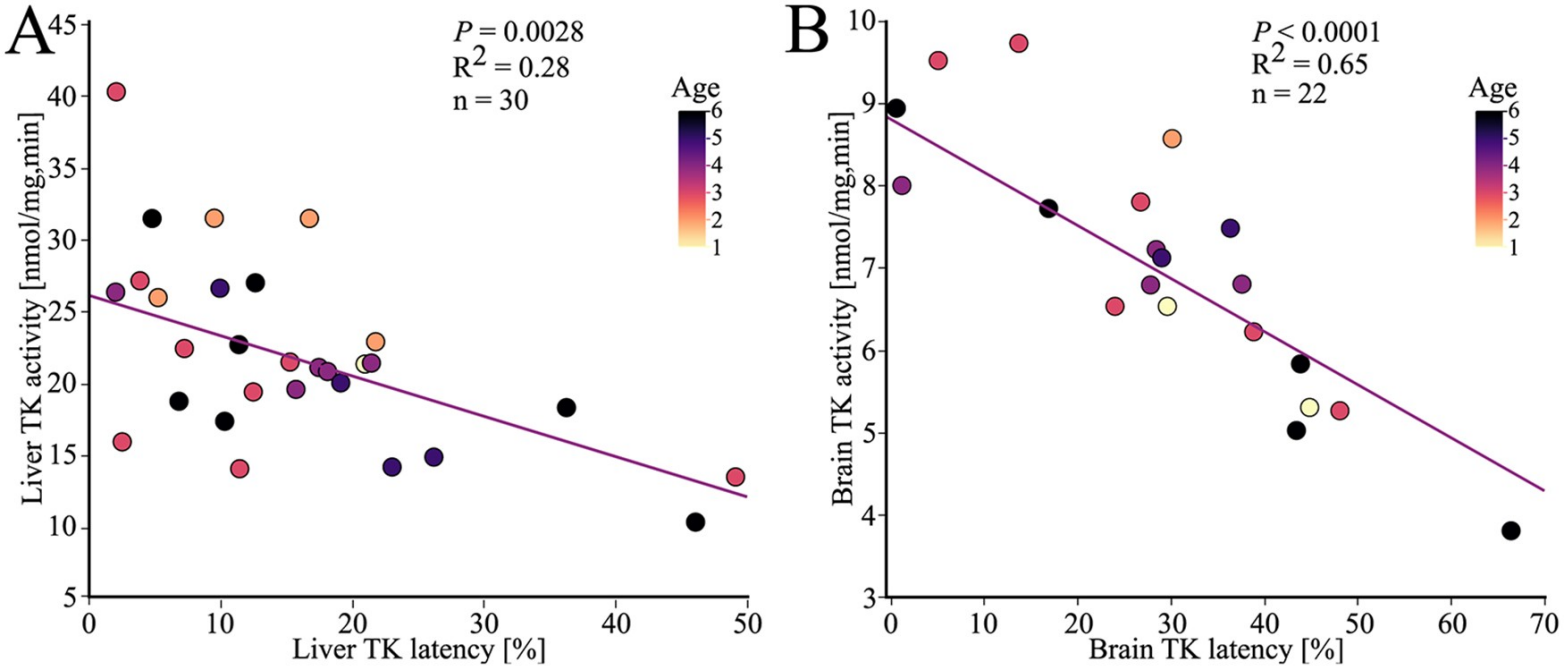
Relationship between TK specific activity and latency, in liver and brain tissue in EB cod. The colors correspond to the age of each specimen, determined by annuli counting of the otoliths. (A) The liver TK activity decreased with increasing liver TK latency. (B) The brain TK activity decreased with increasing brain TK latency.

The low concentration of TDP in the liver in many specimens is reflected in the strong correlation between a decrease of TK activity and increased proportion of TK apoenzymes in the liver, *i*.*e*. high latency (*P* = 0.0028, n = 30, Fig 6A). This is a known correlation observed both among laboratory experimental animals and among wild populations with thiamine deficiency [16, 32, 84]. When the concentration of SumT is low, the proportion of apoenzymes is high. However, the apoenzymes will degrade due to instability [85]. The activity might not be as high as it previously was in a specimen that has suffered from thiamine deficiency for a long period of time [85]. There was a tendency of a correlation between decreased liver TK activity and increasing age (*P* = 0.074, n = 30, represented by colors in Fig 6A) and no correlation between liver TK latency and age (*P* = 0.23, n = 30, represented by colors in Fig 6A). In this study, the correlation between the TK latency and activity is stronger in the brain (*P*<0.0001, n = 22, Fig 6B) compared to the liver (Fig 6A). This is most likely due to better protection against degradation of the apoenzymes in the brain than in the liver, which has been observed previously [86]. Important to note, however, this does not mean that the brain is more thiamine deficient. Due to the fact that the brain is better protected, and thus affected later in the deficiency, no correlation could be seen between increasing age and decreasing activity (*P* = 0.4068, n = 22, represented by colors in Fig 6B) or increasing latency (*P* = 0.3148, n = 22, represented by colors in Fig 6B).

**Fig 7:**
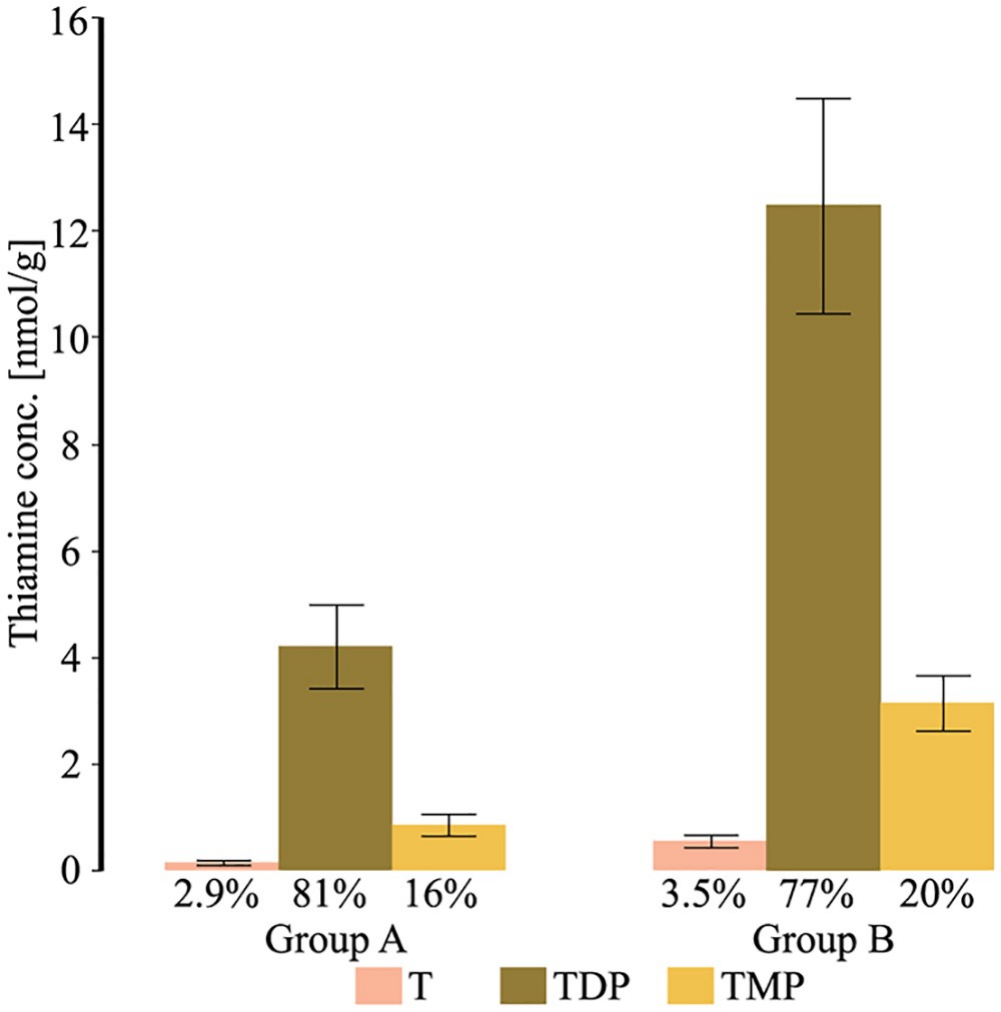
Proportion of T, TDP and TMP in the 15 specimens with the lowest and highest SumT concentrations. The distribution of T, TMP and TDP among the 15 specimens with the lowest SumT concentrations (group A), to the left and the 15 specimens with the highest SumT concentrations (group B), to the right. The proportion of T did not differ between the groups. The proportion of TDP was higher in group A than in group B (*P* = 0.0019). The proportion of TMP was lower in group A than in group B (*P* = 0.0035). Error bars correspond to a 95% confidence interval.

The proportion of the different forms of thiamine can reflect the thiamine status in a tissue. The proportion of T, TMP and TDP were compared between the 15 specimens with the lowest Sum T concentrations (group A) and the 15 specimens with the highest SumT concentrations (group B) (Fig 7). The distribution of the thiamine forms in the lower concentration of SumT, group A (<8 nmol SumT/g liver), was 2.9% T, 81% TDP and 16% TMP while group B (>11 nmol SumT/g liver) had 3.5% T, 77% TDP and 20% TMP. There was no difference between the proportion of T between the groups (*P* = 0.16). However, the proportion of TDP was higher in group A than in group B (*P* = 0.0019). The proportion of TMP was lower in group A than in group B (*P* = 0.0035). It is expected that at low SumT concentrations, the proportion of TDP is kept high to maintain thiamine dependent metabolism, and consequently the proportion of T and TMP are lower than when SumT concentrations are normal [16, 73]. The low concentration of T suggests a normal function of the thiamine pyrophosphokinase, since a thiamine deficient specimen can keep a high proportion of TDP in the cells, a phenomenon that has been previously observed among several wild animals with thiamine deficiency [32]. This indicates that the explanation for thiamine deficiency is not likely due to a malfunction of the enzyme thiamine pyrophosphokinase [32].

**Fig 8:**
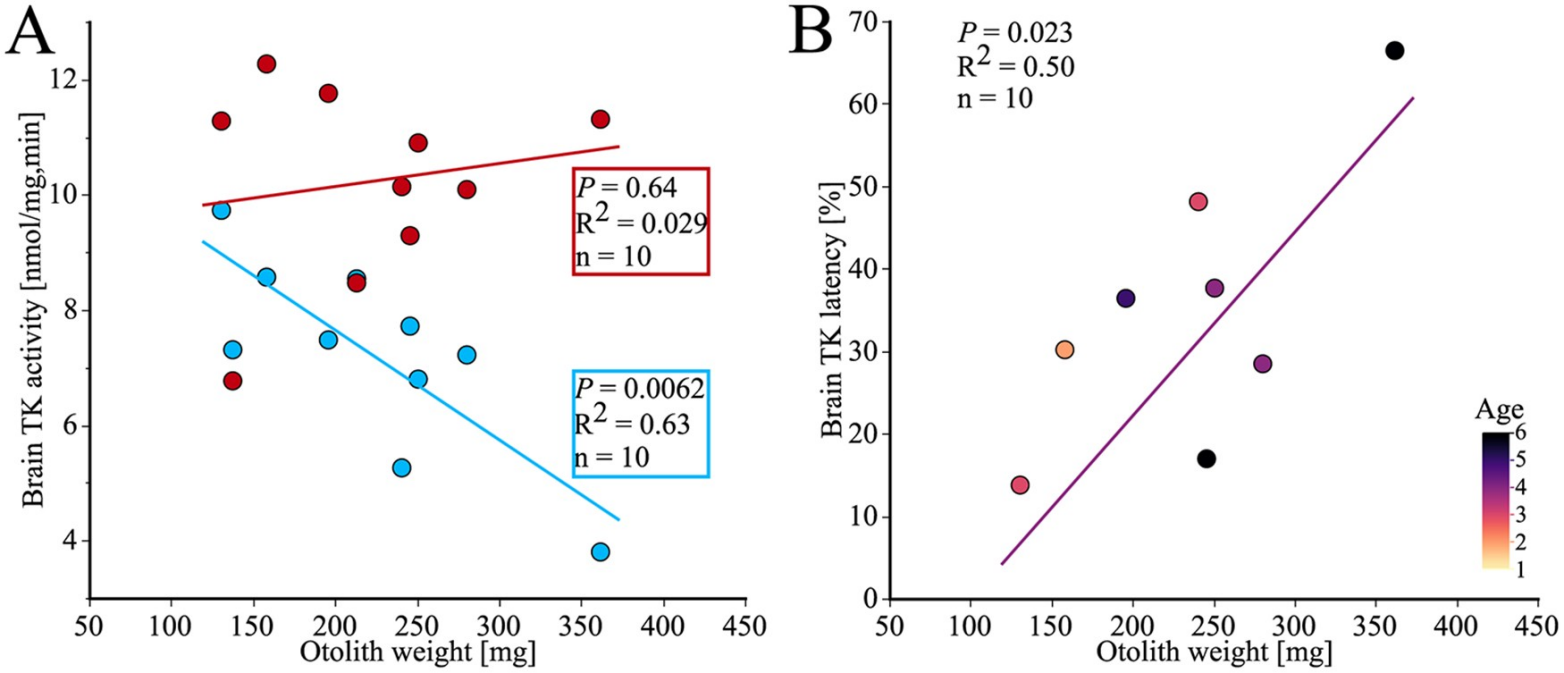
Relationship between otolith weight and TK variables in the brain. (A) The TK endogeneous activity (blue) in the brain decreased with increasing otolith weight while the TK maximum activity (red) was relatively constant with increasing otolith weight. (B) The colors correspond to the age by annuli counting of the otoliths. The brain TK latency increased with increasing otolith weight. Two specimens had latency values below zero (otolith weights 138 mg and 213 mg) and were therefore excluded from the graph, but included in the statistical calculations.

When the somatic weight increased, the liver TK latency increased (*P* = 0.017, n = 30, not shown). When the length of the EB cod increased, the liver TK latency increased (*P* = 0.025, n = 30, not shown). As the liver size increased, the liver TK latency increased as well (*P* = 0.049, n = 30, not shown). When the relative weight of the brain increased, as typically seen in younger fish, the liver TK latency decreased (*P* = 0.021, n = 30, not shown). As the otolith weight increased, the endogeneous TK activity in the brain decreased (*P* = 0.0062, n = 10, blue line Fig 8A), while the maximum TK activity seems to be relatively constant (*P* = 0.64, n = 10, red line Fig 8A). As the otolith weight increased, the brain TK latency in males increased (*P* = 0.023, n = 10, Fig 8B). There was no correlation between the liver TK latency and the otolith weight, a phenomenon that may be based on higher degradation of the apoenzymes in the liver tissue.

**Fig 9:**
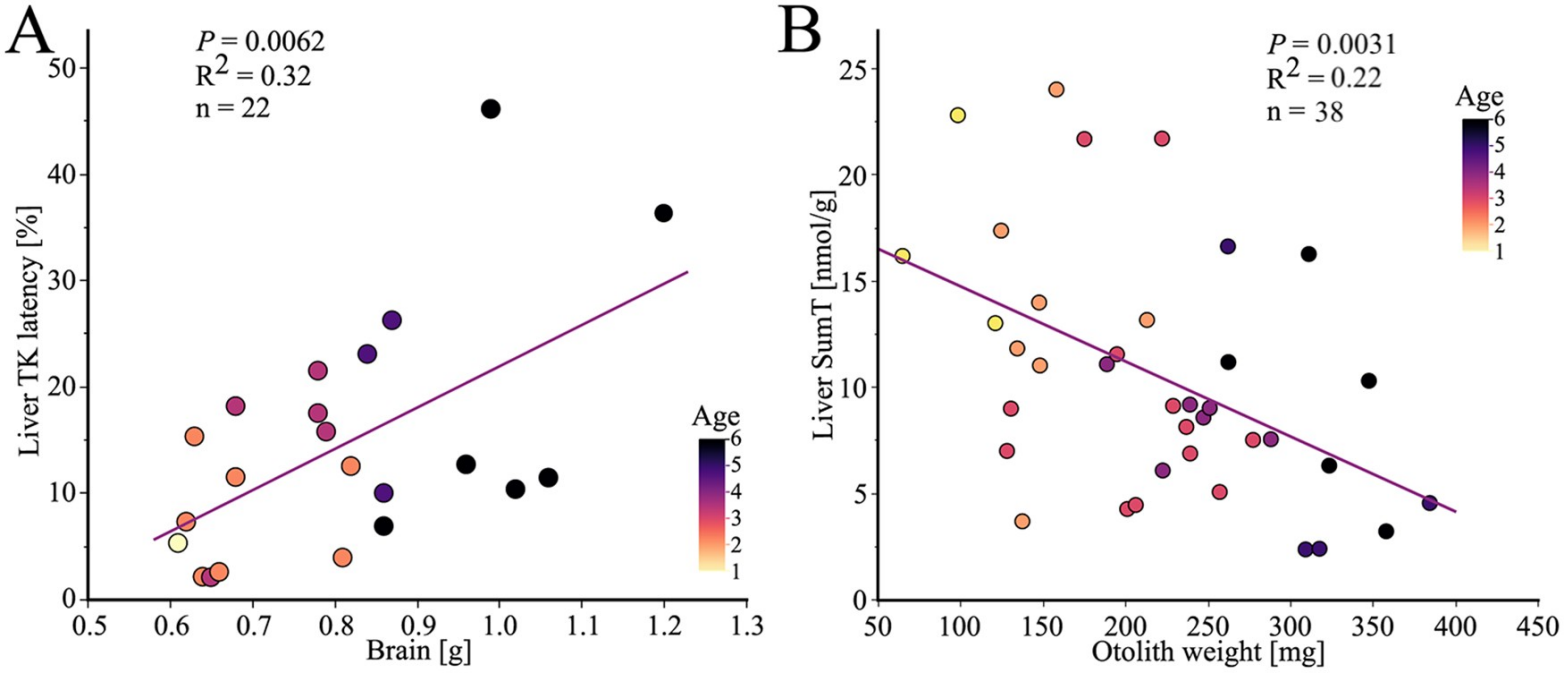
Relationship between thiamine biomarkers and biological parameters. The colors correspond to the age (annuli counting) of each specimen. (A) The liver TK latency increased with increasing weight of the brain when excluding brains smaller than 0.6 g. (B) The liver SumT concentration decreased with increasing otolith weight and age by annuli counting of the otoliths.

There was no correlation between the weight of the brain and the liver TK latency. However, by excluding brains smaller than 0.60 g, mainly corresponding to EB cod younger than 3 years, the liver TK latency increased with increasing brain weight (*P* = 0.0062, n = 22, Fig 9A). The liver SumT concentration decreased with increasing otolith weight (*P* = 0.0031, n = 37, Fig 9B), as well as with the age by annuli counting of the otoliths (*P* = 0.017, n = 37, represented by colors in Fig 9B). This indicates that the thiamine deficiency develops as EB cod get older. Higher demand of thiamine for reproduction and/or lower uptake of thiamine from the food chain cannot be excluded as explanations, given our present state of knowledge.

**Fig 10:**
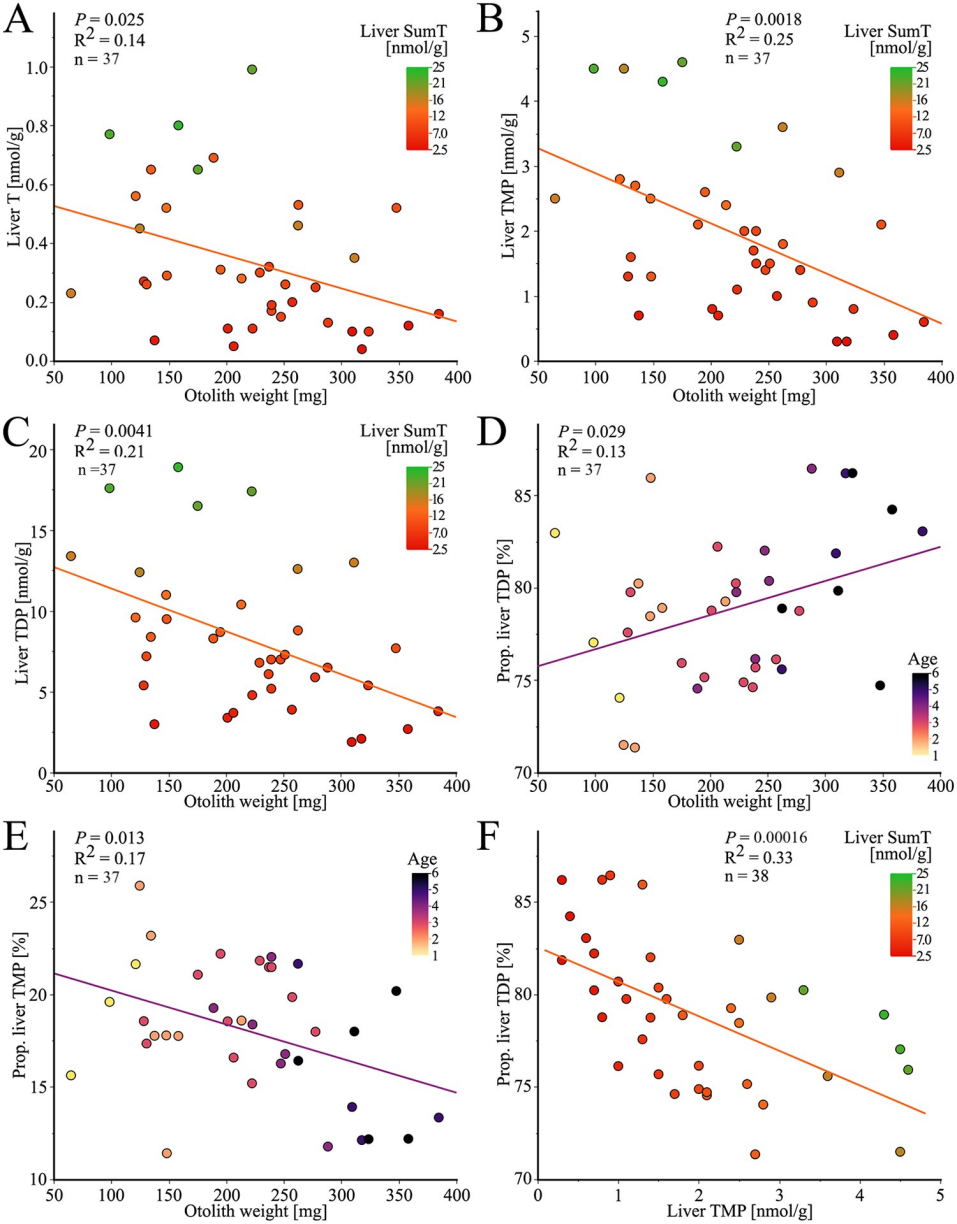
Thiamine concentrations and proportions in the studied EB cod group. Thiamine (T), thiamine monophosphate (TMP) and thiamine diphosphate (TDP) are combind to SumT. (A) The liver T concentration decreased with increasing otolith weight. The colors correspond to the concentration of SumT. (B) The liver TMP concentration decreased with increasing otolith weight. The colors correspond to the the concentration of SumT. (C) The liver TDP concentration decreased with increasing otolith weight. The colors correspond to the concentration of SumT. (D) The proportion TDP (percentage of liver SumT) increased with increasing otolith weight. The colors correspond to the age of each specimen, determined by annuli counting. (E) The proportion TMP (percentage of liver SumT) decreased with increasing otolith weight. The colors correspond to the age of each specimen, determined by annuli counting. (F) The proportion TDP (percentage of liver SumT) decreased with increasing liver TMP concentration. The colors correspond to the concentration of SumT.

Thiamine and its derivatives decreased significantly with increasing otolith weight; T (*P* = 0.025, n = 37), TMP (*P* = 0.0018, n = 37) and TDP (*P* = 0.0041, n = 37) (Fig 10A–10C). The concentration of thiamine and its derivatives decreased with decreasing SumT, T (*P*<0.0001, n = 37), TMP (*P*<0.0001, n = 37) and TDP (*P*<0.0001, n = 37) (represented by colors in Fig 10A–10C). Previous studies have shown that the proportion of TDP increased with severity of thiamine deficiency in specimens with thiamine deficiency [32]. The proportion of liver TDP increased with increasing otolith weight (*P* = 0.029, n = 37, Fig 10D) and increasing total weight (*P* = 0.0095, n = 38, not shown). The proportion of TDP increased with a tendency of significance with the age (*P* = 0.056, n = 37, represented by colors in Fig 10D). This correlation indicates that, as a result of a lower thiamine status, larger and older specimens have the highest proportion of TDP.

The proportion of liver TMP decreased with increasing otolith weight (*P* = 0.013, n = 37, Fig 10E) and increasing total weight (*P* = 0.0033, n = 38, not shown). The proportion of TMP decreased with increasing age (*P* = 0.024, n = 37, represented by colors in Fig 10E), indicating that older specimens might be more thiamine deficient. In a thiamine deficient specimen, there is usually an increase in the proportion of TDP as the TMP concentration decreases [32]. One explanation for this is that the intracellular utilization of TMP increases in specimens with severe thiamine deficiency [32, 87, 88]. This correlation is observed within this EB cod group, the proportion of liver TDP increased with decreasing liver TMP (*P* = 0.00016, n = 38, Fig 10F). The proportion of liver TDP decreased with increasing SumT (*P* = 0.028, n = 38, represented by colors in Fig 10F).

The liver TK latency increased as the liver weight increased (*P* = 0.049, n = 30, not shown), correlating with the chemical analysis (Fig 5A). The correlation could depend on the total weight and/or the age of the EB cod, where older EB cod are more thiamine deficient than younger EB cod. The relative size of the liver does not affect the liver TK latency (*P* = 0.5029, n = 30, not shown). The brain TK latency increased as the SCI in females increased (*P* = 0.00084, n = 12, not shown). In previous studies, the latency decreases as the SCI increases [32]. However, among these EB cod specimens in this correlation between SCI in females and brain TK latency, 83% had a SCI below 0.8, indicating that the thiamine dependent apoenzymes are unstable when the deficiency has prolonged to cause drastic changes to the body condition [85].

The mean liver TK latency was 15 ± 4.5% (range 0–49%, n = 30). The mean brain TK latency was 27 ± 8.3% (range 0–66%, n = 22). The mean liver SumT concentration was 10 ± 1.9 nmol/g (range 2.4–24 nmol/g, n = 38). Due to the instability of the TK apoenzymes in the liver, it is difficult to determine a specific SumT concentration where the EB cod is above the threshold for thiamine deficiency. By combining the chemical and the biochemical analysis, it can be assumed that in a healthy EB cod specimen the SumT concentrations in the liver should at least be in the region of 20 nmol/g or higher. There are many specimens with a concentration half of that, suggesting severe thiamine deficiency in this group. Furthermore, previous analyses of EB cod collected in 1996 showed that female EB cod had liver SumT concentrations below 2 nmol/g and male EB cod had liver SumT concentrations below 4 nmol/g [33], indicating thiamine deficiency in this population more than 20 years ago. These results, combined with the long-term declines seen in EB cod over the past 30 years, raises the question whether the collapse of the EB cod population in the Baltic sea is related to thiamine deficiency. The EB cod seem to be in an even worse state than the Atlantic salmon (*S*. *salar*) and European eel (*A*. *anguilla*), with individual specimens with higher latency in both liver and brain, see Table 1, in addition to the very low levels of SumT in older specimens (Fig 9B).

**Table 1:** Compilation of thiamine status variables in various adult fish species affected by a thiamine deficiency compared with results from this study.

Species	Atlantic salmon (*Salmo salar*) [32]	European eel (*Anguilla anguilla*) [32]	EB cod, spawning (*Gadus morhua*) [33]	EB cod (*Gadus morhua*) (this study)
**Liver SumT** **[nmol/g]**	13±2.1 (n = 22, range 7.1–29)	11.1±1.2 (n = 20, range 5.7–16)	**Females** 1996:2.0±1.4 (n = 10) 1997:1.4±0.77 (n = 9) 1997: 0.98±0.21 (n = 12) **Males** 1996: 4.3±2.6 (n = 2) 1997: 3.8±0.68 (n = 3) (n = 36, range 0.56–4.7)	10±1.9 (n = 38, range 2.4–24)
**Liver TK** **latency [%]**	25±2.1 (n = 22, range 16–33)	7.2±1.9 (n = 20, range 0–15)		15±4.6 (n = 30, range 0–49)
**Brain TK** **latency [%]**	30±3.4 (n = 22, range 5.5–44)	4.7±2.9 (n = 19, range 0–14)		27±8.3 (n = 22, range 0–66)

The data are presented as average ± 95%CI (n, range) and calculated using data from previous work [32, 33].

**Fig 11:**
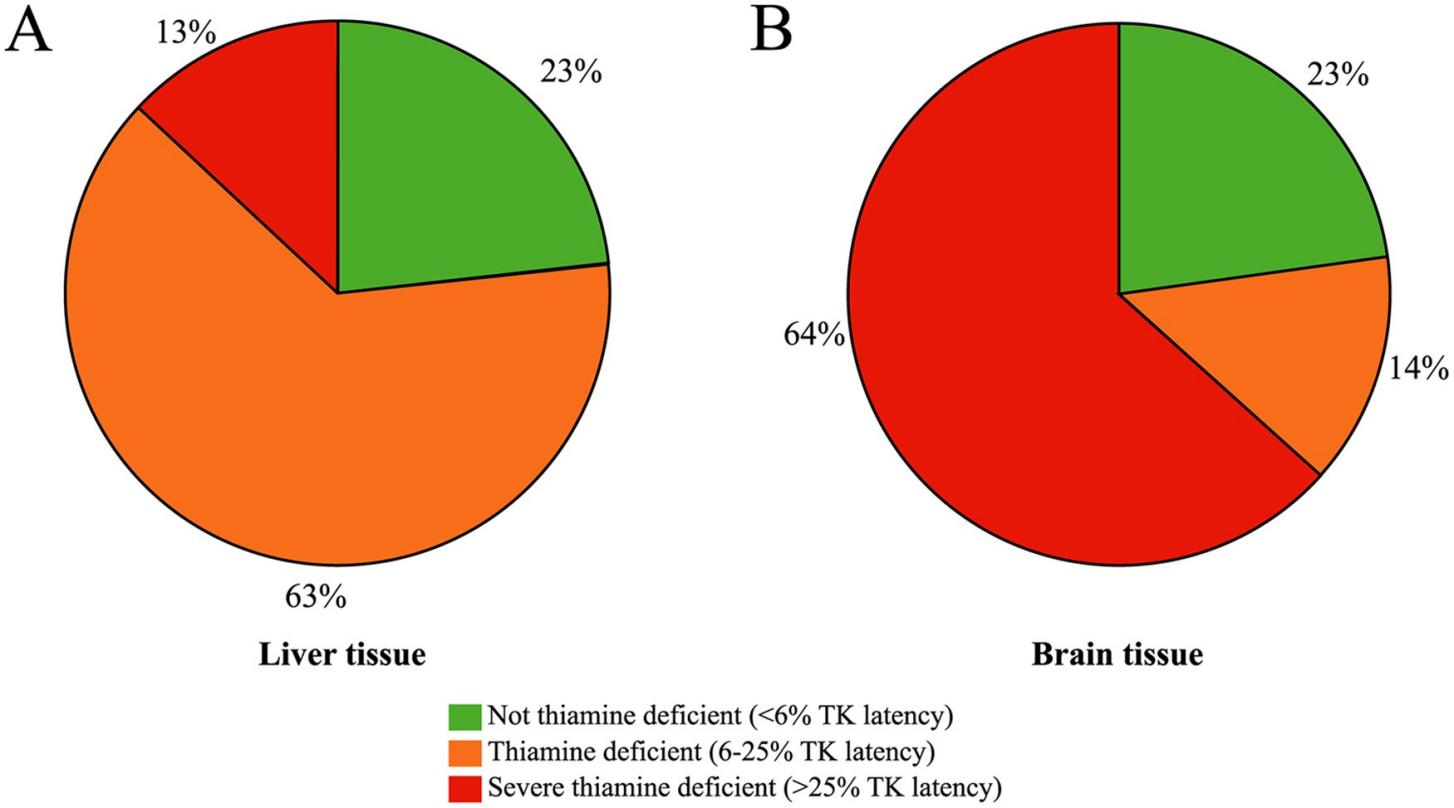
Proportion of the EB cod with no thiamine deficiency, thiamine deficiency and severe thiamine deficiency in liver and brain tissue. Based on an arbitrary definition, non-thiamine deficient tissues were defined as tissues with TK latency <6%, thiamine deficient tissues were defined as tissues with 6–25% latency, and severely thiamine deficient tissues were defined as tissues with more than 25% latency. (A) The liver showed obvious thiamine deficiency in 63% and severe thiamine deficiency in 13% of the EB cod. (B) The brain showed obvious thiamine deficiency in 14% and severe thiamine deficiency in 64% of the EB cod.

The analyzed liver tissue showed that 76% of the EB cod had thiamine deficiency in the liver (Fig 11A) with an average of 19% liver TK latency (n = 23, not shown) among the thiamine deficient specimens. The analyzed brain tissue showed that 78% of the EB cod had thiamine deficiency in the brain (Fig 11B) with an average of 34% brain TK latency (n = 17, not shown) among the thiamine deficient specimens. These measurements showed that 13.3% of the livers and 63.6% of the brains where severely thiamine deficient (latency >25%) (Fig 11A and 11B). At first sight, this could give the impression that the brain is more affected than the liver of the ongoing thiamine deficiency. However, our interpretation is that the TK apoenzyme might be more stable in the brain tissue than in the liver tissue, resulting in higher latency values in the brain (Fig 6A and 6B). Even though this study only measured the SumT concentration in the liver, the fact that a decline of SumT is often more pronounced in the liver than in the brain, is an established phenomenon among different species where thiamine deficiency has developed over time [17, 16, 32, 89, 90].

The summarized results from this study are compared with European eel, Atlantic salmon [32] and results from the previous study in 1996 [33] regarding EB cod in Table 1. In fact, among the EB cod in this study, there were a few specimens that had up to ten times lower SumT concentrations compared to others, also suggesting that the analyzed EB cod group contains specimens that have severe thiamine deficiency.

A declining cod population was observed in Newfoundland in the 1980s, and in 1992 all commercial fishing of Atlantic cod in the area was banned [91]. The explanations for the disappearance of the Atlantic cod in that area was over-fishing, the same as one of the major hypotheses for the decline in the Baltic Sea today. However, the Newfoundland Atlantic cod population has not recovered since then, despite large reductions of fishing pressure [92]. Other observations included smaller fish [93], early maturation [93, 94], lower body condition [95, 96], decline of energy reserves [96] and skipped spawning [97]. The cod population in Newfoundland had a decreasing CF just like the EB cod population today [98]. The fact that the population has not recovered has led to a change in speculation for the decline, to "elevated natural mortality" [94]. However, these symptoms are not inconsistent with thiamine deficiency, and appear similar to what we see currently happening to the EB cod. To our knowledge, the concentration of thiamine and the TDP-dependent enzymes has not been investigated in the Atlantic cod population in the waters outside Newfoundland, and it cannot be ruled out that the population might suffer from thiamine deficiency in this region. In 1958, the average CF in the Newfoundland cod population was varying around a mean of 1, and decreased to around 0.85 in 1993 [95]. It seems that the cod in Newfoundland die around a CF value of 0.4 [95], and this could explain the lack of specimens below 0.6 in our study. Toxic compounds such as glyoxals and lactic acid may reach lethal values in these species [18, 19]. The liver SumT concentration in the EB cod population could be assumed to be lower in adult tissues in connection with gonad development and egg maturation. Because this study sampled EB cod about 6 months prior to maturation of the eggs, lower levels of thiamine in the adult tissues could be expected closer to the spawning period. In fact, this difference in sampling period compared to the previous study in 1999 may at least partly explain their even lower SumT levels in the liver [33]. While compiling results from across years suggests that the wild EB cod population is thiamine deficient during the entire year, greater temporal resolution is required to determine whether this might be the case.

Results in 1994 indicated that the reproductive success of EB cod was impaired, and that there were increases in mortality and disorders among the offspring correlated to the female, similar to the effects seen in the offspring of thiamine deficient Baltic salmon [15]. A previous study in 1999 argued that the reproductive failure and population decline of the EB cod was not due to M74 [99], which is an old, partly misleading term for thiamine deficiency in salmon offspring [32]. However, the conclusion was drawn based on the comparison of the concentration of thiamine in eggs in Atlantic salmon, incorrectly assumed healthy, compared to the EB cod gonad concentrations [33]. Today we know that these SumT concentrations were too low to produce healthy offspring [32]. In fact, the authors from the work in 1999, who performed the chemical analytical work, concluded that they were not able to determine the thiamine status in EB cod at the time of publication [33]. Fish are affected by thiamine deficiency during embryonic and larval development, because the thiamine deficient adult female is not able to transport the necessary amount of thiamine to the maturating eggs [100]. Larvae with a low thiamine level can therefore be assumed to die in the early life stages. The adult EB cod are affected by thiamine deficiency, and might die directly, as a result of glyoxals, lactic acid, phytanic acid and/or neurological disturbances. However, death probably more commonly occurs as a consequence of secondary disorders of the deficiency, such as orientation problems, weakened senses and/or immunosuppression leading to infections of bacteria, virus, fungi and/or parasites [32]. Previous studies have shown that certain animals become anorectic and emaciated during thiamine deficiency [16]. Furthermore, it has also been shown that starvation does not lead to an increase in latency, simply due to the fact that when a specimen is starving, it does not eat, and does not need to metabolize any food, and therefore the specimen needs less thiamine [16, 84]. Thus, the fact that the CFs of the EB cod are low, cannot explain the thiamine deficiency, although, the thiamine deficiency could explain the low CFs.

The increase of the infestations and prevalence of nematodes in the EB cod could be due to a thiamine deficiency leading to immunosuppression. For example, rats with thiamine deficiency are more susceptible to different parasitic infections, including nematode infections [101]. Furthermore, the thiamine deficient European silver eels and American eels, are infected by the nematode *Anguillicola crassus*, and the prevalence has increased throughout the assumed time period of thiamine deficiency and during the species decline in the last decades [32].

As a final remark, the low growth, low body condition, high mortality, altered metabolism, survival of offspring, emaciation and parasite infections of the EB cod, stated in the recently published ICES report [43], are all common and expected symptoms of the thiamine deficiency that is present among the EB cod.

## Conclusion

The relative proportion of the different phosphorylated thiamine forms in the liver strongly indicate that the studied eastern Baltic cod are thiamine deficient. This is supported by the extremely low concentrations of SumT in the liver. Thiamine deficiency is also indicaded by severely decreased thiamine levels in the liver in older easter Baltic cod. The fact that these levels were also observed more than 20 years ago might suggest that the thiamine deficiency has continued for many years in the Baltic Sea, in concordance with many other thiamine deficiency-affected species in the area [32]. The strong negative correlation between specific endogenous transketolase activity and the proportion of transketolase apoenzymes clearly suggests thiamine deficiency in the liver tissue. The even stronger negative correlations between specific endogenous transketolase activity and the proportion of transketolase apoenzymes in brain tissue further demonstrates thiamine deficiency in the studied eastern Baltic cod. The occurrence of eastern Baltic cod specimens with latency above 45% in both liver and brain, suggests an alarming and continuing thiamine deficiency in this group. Furthermore, to support this on-going thiamine deficiency in eastern Baltic cod, the expected clinical signs of thiamine deficiency have been observed for many years now, such as reduced reproduction, emaciation, secondary infections from fungi, bacteria and parasites, increased mortality, and reduced growth resulting in an very low body condition factor. Overall, the ongoing thiamine deficiency among EB cod, and the secondary effects that are a consequence of the deficiency, point to the important research that is needed, to determine the causative agent(s) and the biochemical mechanism(s) behind this environmental disturbance.

## Supporting information

S1 Table Compiled data. (XLSX)
